# Photoisomerization detected in a fully wavelength-tunable rhodopsin mimic system

**DOI:** 10.1107/S2059798326003839

**Published:** 2026-05-27

**Authors:** Nona Ehyaei, Courtney Bingham, Katelyn Silva, Zahra Nossoni, Hadi Nayebi Gavgani, Meisam Nosrati, Joelle Eaves, Mustapha Akhdar, Chrysoula Vasileiou, Babak Borhan, James H. Geiger

**Affiliations:** a Department of Chemistry Michigan State University East Lansing MI48824 USA; Oak Ridge National Laboratory, USA

**Keywords:** protein design, rhodopsin mimic, single-crystal photolysis, bacteriorhodopsin, protein electrostatics

## Abstract

We describe the photoisomerization of the retinylidene protonated Schiff base in human retinol-binding protein II (hCRBPII) and the role of water molecules in this process. We characterize the photoisomerization of the 15-*cis*/all-*trans* retinylidene protonated Schiff base in this system using UV–visible spectroscopy and atomic-resolution X-ray crystallography. We further demonstrate a process where the p*K*_a_ of the protonated Schiff base is substantially altered by light-induced dehydration of the binding pocket, suggesting novel pathways of photoswitching that rely not on isomerization or conformational change of the chromophore but rather on light-induced reorganization of the protein environment.

## Abstract 2

The mechanism of retinal photoswitching is illuminated in a designed rhodopsin mimic. Two mechanisms, a conventional *cis*–*trans* isomerization and an unprecedented ‘photodehydration’, are observed at atomic resolution in single crystals.

## 1. Introduction

The rhodopsin protein family is a large family of integral membrane proteins. They perform a variety of functions, including as light-activated ion channels and light-driven photosynthetic proton pumps and in signaling, sensing and vision (Filipek *et al.*, 2003 ▸; Kandori, 2020 ▸). Microbial and animal rhodopsins typically bind all-*trans*- or 13-*cis*-retinal, respectively, and both undergo *cis*/*trans* photoisomerization of double bonds, often on a femtosecond timescale (Filipek *et al.*, 2003 ▸; Kandori, 2020 ▸; Spudich *et al.*, 2000 ▸; Tsai *et al.*, 2018 ▸; de Grip & Ganapathy, 2022 ▸; Rozenberg *et al.*, 2021 ▸). Further studies suggest that the isomerization between *cis* and *trans* forms can expose the Schiff-base N atom to distinct protein environments, which in turn leads to distinct p*K*_a_ values for each iminium form. In microbial rhodopsin this isomerization results in a 5–6-unit p*K*_a_ change, and in the rhodopsin visual pigment this difference is as high as 8–11 units (Ernst *et al.*, 2014 ▸; Tsai *et al.*, 2018 ▸). The substantial difference in p*K*_a_ is likely to be the result of distinct electrostatic microenvironments within the protein binding cavity. The ability of proteins to produce the electrostatic fields required for this phenomenon has been extensively studied by the Warshal and Boxer laboratories, most notably in the context of enzyme reactivity (Warshel, 1981 ▸; Fried & Boxer, 2017 ▸; Warshel *et al.*, 1989 ▸, 2006 ▸; Warshel & Levitt, 1976 ▸; Zheng *et al.*, 2022 ▸; Ji & Boxer, 2022 ▸). Although the photoisomerization in bacteriorhodopsin has been studied using NMR, Raman, FTIR, X-ray crystallo­graphy, cryo-EM, XFEL time-resolved crystallography and theoretical calculations (Hayashi *et al.*, 2001 ▸; Kaur *et al.*, 2019 ▸; Lanyi, 2004 ▸; Nogly *et al.*, 2018 ▸; Rothschild, 1992 ▸), less is known regarding the nature of the local electrostatics in these systems.

Over the past two decades, we have investigated a variety of rhodopsin mimics to understand ground-state behavior, absorption tuning and various photochemical states (Ghanbarpour, Nairat *et al.*, 2019 ▸; Nosrati *et al.*, 2016 ▸; Berbasova *et al.*, 2016 ▸; Yapici *et al.*, 2015 ▸). To this end we have been using intracellular lipid-binding protein family members, specifically human cellular retinoic acid-binding protein II (hCRABPII) and human cellular retinol-binding protein II (hCRBPII), as structural templates for the design, construction, investigation and manipulation of rhodopsin mimics, and have generated new fluorescent and allosteric proteins (Maity *et al.*, 2023 ▸; Santos *et al.*, 2020 ▸, 2021 ▸; Demoulin *et al.*, 2021 ▸; Sheng *et al.*, 2020 ▸; Manathunga *et al.*, 2020 ▸; Ghanbarpour *et al.*, 2020 ▸; Ghanbarpour, Pinger *et al.*, 2019 ▸; Ghanbarpour, Nairat *et al.*, 2019 ▸; Sheng, Nick *et al.*, 2018 ▸; Sheng, Nairat *et al.*, 2018 ▸; Berbasova *et al.*, 2013 ▸, 2016 ▸, 2018 ▸; Nosrati *et al.*, 2016 ▸; Assar *et al.*, 2016 ▸; Yapici *et al.*, 2015 ▸; Wang *et al.*, 2012 ▸, 2014 ▸; Huntress *et al.*, 2013 ▸; Vasileiou, Wang *et al.*, 2009 ▸; Vasileiou, Lee *et al.*, 2009 ▸; Vaezeslami *et al.*, 2006 ▸, 2008 ▸; Vasileiou *et al.*, 2007 ▸; Crist *et al.*, 2006 ▸).

These proteins are attractive due to their ease of high-resolution structural analysis, their robustness and their relative insensitivity to mutation. They also possess a relatively large internal binding cavity whose properties are easily manipulated by mutation (Berbasova *et al.*, 2016 ▸; Lee *et al.*, 2021 ▸). We have created mimics that recapitulate the wavelength regulation observed in pigmented rhodopsins (Wang *et al.*, 2012 ▸, 2014 ▸; Berbasova *et al.*, 2013 ▸), along with mutants that exhibit specific photoisomerization cycles that result in substantial changes in iminium p*K*_a_ (Ghanbarpour, Nairat *et al.*, 2019 ▸; Nosrati *et al.*, 2016 ▸), the two basic biophysical characteristics of a rhodopsin mimic. Previously, we have created two distinct rhodopsin photoisomerization mimics using the hCRABPII protein design template: one a 15-*cis* to 15-*trans* photoisomerization, and one an all-*trans* to 15-*cis*-13-*cis* photoisomerization more similar to bona fide microbial rhodopsins, with both processes observed in single crystals at atomic resolution (Ghanbarpour, Pinger *et al.*, 2019 ▸; Nosrati *et al.*, 2016 ▸).

Herein, we delve more deeply into the photoisomerization of the hCRBPII system. We first demonstrate 15-*cis*/all-*trans* retinylidene photoisomerization in both solution and single crystals. Furthermore, we report two different environments with varying p*K*_a_ values for the 15-*trans* isomer. We hypothesize that exposure to visible light drives the protein environment to an even lower p*K*_a_ environment for the iminium by altering the dispensation of ordered water molecules in the binding pocket. We characterized this process using atomic-resolution crystallography and UV–Vis spectroscopy. This photoswitchable rhodopsin mimic can be used as another template for the study of the photophysics of rhodopsin and for the design of new photoswitchable fluorescent protein tags.

## 2. Materials and methods

### 2.1. Materials

Bacterial cells and cell lysates were collected by centrifugation with a Sorval RC-5B temperature-regulated centrifuge from Du Pont Instruments. Cells were lysed by sonication with an ultrasonic homogenizer from Biologics Inc. A Pharmacia Biotech FPLC system was used for protein purification. Fast Q and Source Q anion-exchange resins used for protein purification were purchased from GE Healthcare, as was Superdex 75 resin for purification by size exclusion. UV–Vis measurements were performed using a Cary 300 BioWin UV spectrophotometer (Varian Instruments). All chemicals were purchased from Sigma–Aldrich and were used without further purification or modification unless otherwise specified.

### 2.2. Site-directed mutagenesis

The hCRBPII-pET-17b plasmid described previously was used for mutagenesis using the Q5 Site-Directed Mutagenesis Kit protocol (New England Biolabs). PCR conditions for the amplification of mutants are specified in Tables 1 ▸, 2 ▸ and 3 ▸.

### 2.3. Expression of hCRBPII mutants

The target gene (1 µl at ∼100 ng µl^−1^) was transformed into 50 µl of chemically competent *Escherichia coli* BL21(DE3) pLysS cells. The sample was kept on ice for 30 min, submerged in water equilibrated to 42°C for a 10 s heat shock and then returned to ice for an additional 5 min. The sample was then grown in 950 µl lysogeny broth (LB; 1 ml total solution) at 37°C for a minimum of 1 h, after which 200 µl sample was spread on an agar plate augmented with ampicillin (100 µg ml^−1^). The agar plate was then incubated at 37°C overnight. A single colony of the transformed cells was used to inoculate 1 l LB containing 1 ml 100 µg ml^−1^ ampicillin. The inoculated culture was allowed to shake at 37°C for approximately 9 h, until the OD_600_ reached 0.6–0.8. Protein overexpression was initiated by adding 1 ml 1 m*M* isopropyl-β-d-1-thiogalactopyranoside (IPTG; Gold Biotechnology). The 1 l culture was then transferred to a shaker equilibrated at 23°C and allowed to shake for 20 h.

### 2.4. Protein purification

The cells were collected by centrifugation (5000 rev min^−1^ for 20 min) and resuspended in 100 ml lysis buffer (10 m*M* Tris–HCl pH 8.0). The cell resuspension was lysed by sonication (1 min, 80% power, ×3) and the cell lysate was then spun down (10 000 rev min^−1^ for 30 min). The supernatant was deposited onto Q Sepharose Fast Flow anion-exchange resin for protein to bind by gravity flow. The resin was then washed with 100 ml lysis buffer. Protein was eluted with 100 ml elution buffer (10 m*M* Tris–HCl, 150 m*M* NaCl pH 8.0) and the eluent was desalted with lysis buffer using Amicon Ultra centrifugal filter units (15 ml, 10 kDa cutoff). This buffer exchange was performed by three rounds of 30 min centrifugation at 5000 rev min^−1^. The desalted sample was then further purified by anion exchange using a Pharmacia Biotech FPLC system equipped with a Source 15Q anion-exchange column according to the program given in Table 4 ▸.

Fractions were analyzed for purity by SDS–PAGE. Clean fractions were then collected, concentrated to a volume below 3 ml and purified by size-exclusion chromatography using a Pharmacia Biotech FPLC system equipped with a Superdex 75 gel-filtration column. Protein fractions were taken and assessed for purity by SDS–PAGE.

### 2.5. Protein crystallization

Pure protein samples were concentrated to 10–15 mg ml^−1^ using Amicon Ultra centrifugal units (5 ml, 10 kDa cutoff), and ∼4 equivalents of all-*trans* retinal were added in the dark. Crystallization was performed by vapor diffusion using 24-well plates (purchased from Hampton Research) with 1 ml reservoir volume. For each well, 1 µl protein solution was added to 1 µl well solution. Crystals typically appeared within 1–3 days in well solutions consisting of 25% PEG 4000, 0.1 *M* ammonium acetate, 0.1 *M* sodium acetate with a pH range of 4.0–4.8. Crystals were flash-cooled in a solution consisting of the mother liquor and 18–20% glycerol.

### 2.6. UV–Vis measurements and light irradiation

UV–Vis measurements were made in phosphate-buffered saline adjusted to pH 4 using a citric acid solution. All UV irradiation was performed using a TLC UV hand lamp. Samples were exposed to UV light for long enough to ensure total conversion of the Schiff base (SB) to protonated Schiff base (PSB). In solution, this conversion was monitored by the disappearance of the SB peak at 360 nm and growth of the PSB peak at 612 nm in the absorbance spectrum. The progress of SB–PSB photoconversion was monitored in the crystal by the change in color from colorless to blue. Likewise, conversion back to the SB was monitored by leaving the sample under white light until the visual cues described above indicated that the sample had completely converted to the SB form.

### 2.7. Crystallographic data collection and refinement

Diffraction data were collected at LS-CAT sector 21 (21-ID-D, 21-ID-F and 21-ID-G) at the Advanced Photon Source (APS), Argonne, Illinois, USA using 1.127 Å wavelength radiation at 100 K using either an EIGER 9M, MAR 300 or MAR 350 detector. Data reduction and scaling were performed using the *HKL*-2000 program package (Otwinowski & Minor, 1997 ▸). All structures were solved by molecular replacement using *Phaser* in *Phenix* and were refined using the *Phenix* program package (Liebschner *et al.*, 2019 ▸). Three cycles of refinement were implemented for each run. Placement of the retinal ligand and all ordered water molecules was performed using *Coot* (version 0.8.9.1; Emsley *et al.*, 2010 ▸). The statistics associated with crystallographic data collection and refinement are given in Table 5 ▸.

## 3. Results and discussion

### 3.1. The ‘reference’ binding pocket of retinylidene-bound hCRBPII for isomerization

**Figure 1:**
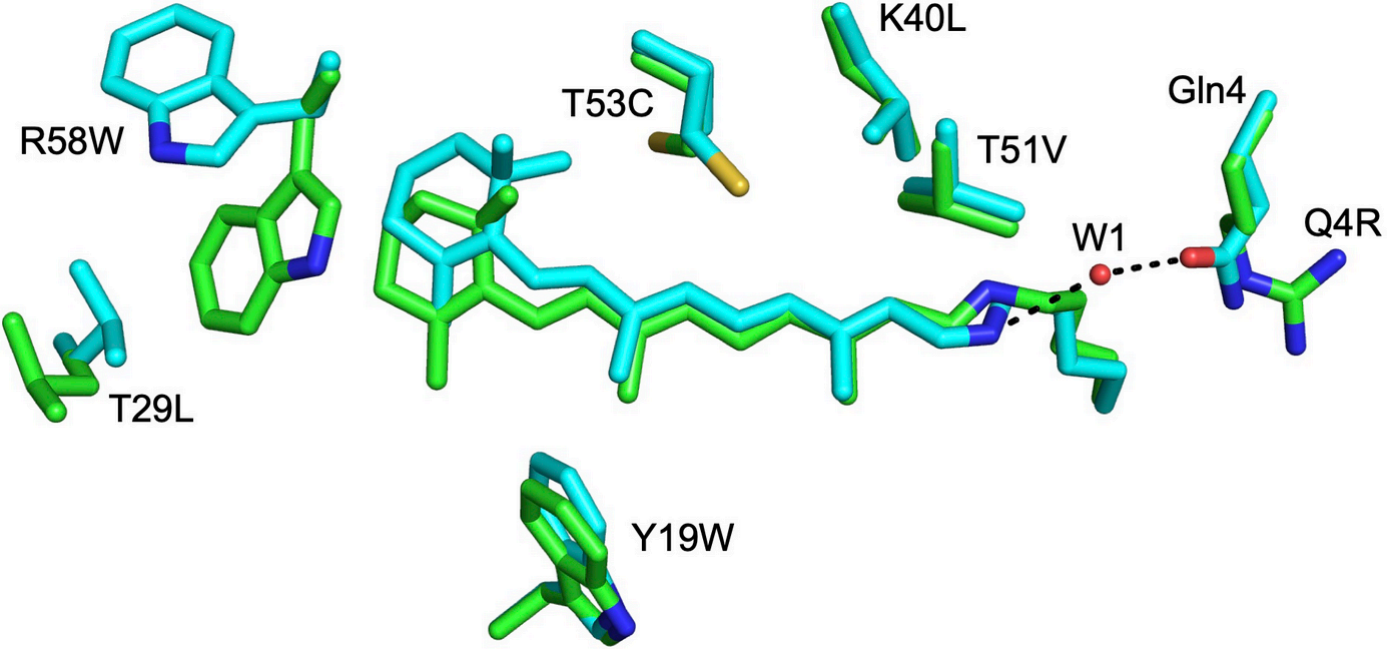
The structures of the Q108K:K40L:T51V:T53C:R58W:Y19W:T29L (cyan C atoms, λ_max_ = 591 nm, PDB entry 4efg) and Q108K:K40L:T51V:T53C:R58W:Y19W:T29L:Q4R (green C atoms, λ_max_ = 622 nm, PDB entry 4eej) mutants overlaid. Mutation of Gln4 leads to loss of the ordered water molecule that helps stabilize the *cis*-isomer, resulting in the retinylidene 15-*trans* conformation. All other atoms are colored by type here and in all subsequent figures (N, blue; O, red; S, yellow).

Two mutations in hCRBPII, Q108K and K40L (KL), are required for protonated Schiff-base (PSB) formation (Wang *et al.*, 2012 ▸, 2014 ▸). The structures of retinal-bound complexes obtained for the overwhelming majority of hCRBPII variants indicate the PSB linkage between protein and retinal to be the 15-*cis* isomer, despite the fact that it is expected to be thermodynamically disfavored relative to the *trans* isomer. In virtually all cases the *cis* isomer appears to be stabilized by the combination of an NH–π interaction between the iminium and Trp106 and a water-mediated interaction between the iminium and Gln4 (Supplementary Fig. S1; Wang *et al.*, 2012 ▸). In contrast, structures of Gln4 mutants have invariably shown a *trans*-imine conformation. The overlaid structures of Gln4 mutants with structures that retain Gln4 show the loss of the ordered water molecule that helps to stabilize the *cis* isomer, indicating that the loss of the water-mediated interaction destabilizes the *cis* isomer, resulting in the *trans* isomer (Fig. 1 ▸).

Spectroscopic experiments indicated that Gln4 mutants were also prone to loss of Schiff-base protonation over time, indicating a conversion to a more thermally stable neutral form of the retinylidene (Wang *et al.*, 2012 ▸). This behavior is similar to that seen in hCRABPII, where the loss of Schiff-base protonation was demonstrated to be the result of *cis*/*trans* isomerization of the imine bond (Nosrati *et al.*, 2016 ▸). It is important to note here that although Trp106 is conserved in hCRABPII, Gln4 is not, with the equivalent position occupied by a Phe residue. Given this precedent, we hypothesized that a similar isomerization may be occurring in hCRBPII Gln4 mutants, where the *cis*-iminium, which is initially formed in the reaction between the chromophore and Lys108, thermally isomerizes over time to the *trans*-imine isomer, with concomitant loss of Schiff-base protonation. Consistent with this hypothesis are the structures of hCRBPII variants [see, for example, PDB entries 4ruu, 4efg and 4ede (Supplementary Fig. S1) versus 4gkc and 4eej] (Wang *et al.*, 2012 ▸). While the *cis*-isomers invariably show the NH–π interaction with Trp106 stabilizing the PSB, the *trans* isomers show the Schiff-base N atom to be surrounded by hydrophobic residues, with K40L, Leu117, Leu115 and Ile42 sitting within ∼5 Å of the iminium nitrogen (Supplementary Fig. S2). This is consistent with a low-p*K*_a_ environment that would give rise to deprotonation of the iminium. If true, it is expected that *cis*/*trans* photoswitching is possible in hCRBPII, just as was seen in hCRABPII variants. To explore this, a number of Gln4 mutants were explored, culminating in the investigation of the Q108K:K40L:T51V:T53C:Y19W:R58W:T29L:Q4A (**M1**) mutant, which has the *trans*-iminium structure and is one of the most red-shifted mutants of the hCRBPII system (Wang *et al.*, 2012 ▸).

### 3.2. Thermal switching and photoswitching of hCRBPII variants in solution

**Figure 2:**
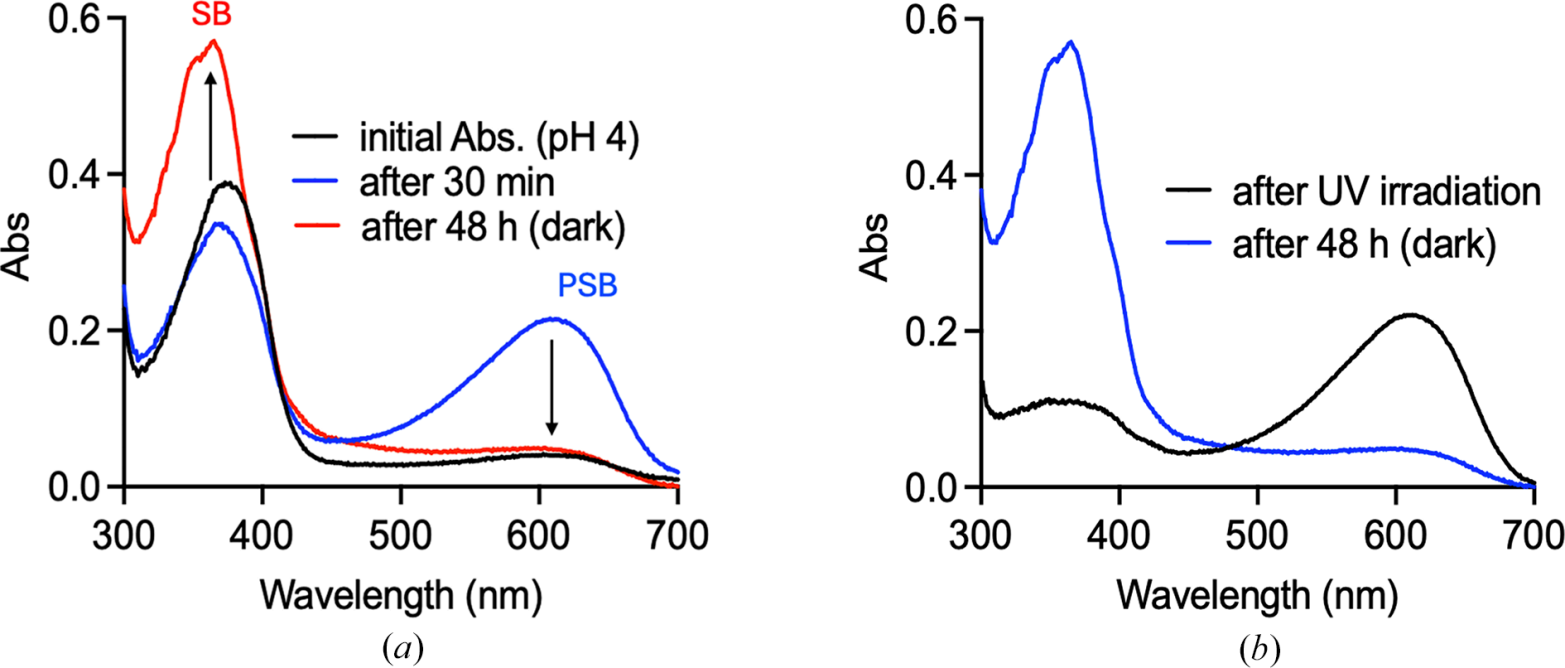
(*a*) Absorption spectra of retinal-bound M1 at pH 4 monitored over time. Maximum PSB is detected after 30 min (blue line). The PSB is not stable and thermally converts to SB over time (red line, two days). (*b*) Incubation of **M1** with retinal for 48 h (blue line). UV light irradiation converts the Schiff base to PSB (black line). Note the residual PSB absorption even after 48 h.

Initially, hCRBPII variants were investigated spectroscopically in solution. Addition of retinal to hCRBPII variants with the Q108K:K40L mutations resulted in the formation of retinylidene PSB complexes, identified by the characteristic absorbance in the visible spectrum, which is tunable by various mutations in the binding pocket. This initial PSB formation was observed in each mutant regardless of the presence or absence of a Gln4 mutation (Wang *et al.*, 2012 ▸). However, while most mutants with Gln4 form a PSB that is relatively stable over time, variants in which Gln4 is mutated to an aprotic, hydrophobic residue lost their absorption in the visible portion of the spectrum and increased their absorption at 360 nm. This is characteristic of the Schiff-base absorption, the wavelength of which is mostly independent of protein environment. An example of this phenomenon is shown for **M1** (Fig. 2 ▸*a*), where PSB formation occurs over 30 min and then decays over several hours. This was observed at both a pH of 7 (Supplementary Fig. S3) and a pH of 4, the latter coinciding with the pH of crystallization for most hCRBPII variants. Titration of the initial PSB form gives a p*K*_a_ of ∼7 (Supplementary Fig. S4). Acidification of the complex obtained after 24 h (the SB form) leads to protonation of the Schiff base, but with a p*K*_a_ of 3.3 (Supplementary Fig. S4). This suggests a thermal conversion of the PSB from a higher to lower p*K*_a_ form, similar to that seen in our previous hCRABPII system (Fig. 2 ▸*b*; Nosrati *et al.*, 2016 ▸).

We next investigated the possibility of photoswitching in this system. To this end, retinylidene-bound **M1** was incubated in the dark for 24 h at pH 7, resulting in minimal absorption in the visible and maximal absorption at 360 nm (Supplementary Fig. S3). Irradiation with UV light resulted in a loss of UV absorption and a concomitant increase in the absorption at 612 nm, consistent with photoconversion of the SB to the PSB. The same conversion was also observed at pH 4, indicating that the complex can be photoswitched between SB and PSB forms at either pH (Fig. 2 ▸*c*). Under both pH conditions, irradiation of the resulting complex with visible light reversed the process, converting the visible light-absorbing PSB to SB (Supplementary Fig. S3).

### 3.3. Elucidation of the hCRBPII photoswitching mechanism in single crystals

**Figure 3:**
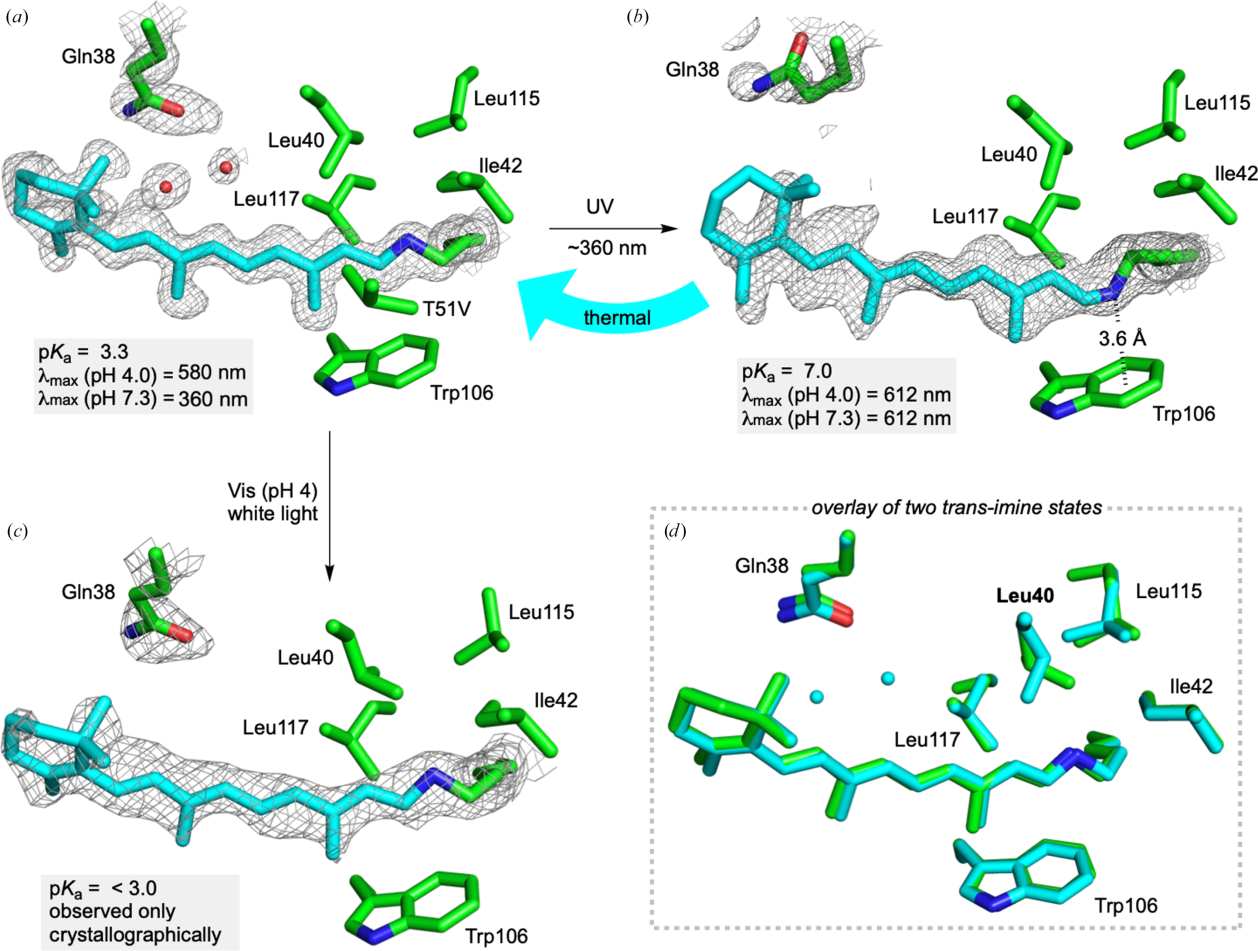
(*a*) Structure of the retinal-binding pocket in the blue-tinted **M1** crystal (grown in the dark, 1.40 Å resolution). The imine nitrogen is surrounded by hydrophobic interactions consistent with its lower p*K*_a_ of 3.3. Protein residues are colored by atom type, with C atoms in green. The retinal chromophore is cyan. (*b*) UV exposure of the **M1** crystals give the structure of the dark blue crystal with the 15-*cis* iminium (1.26 Å resolution). The positive charge on the iminium is stabilized by the NH–π interaction with Trp106 [colors as in (*a*)]. (*c*) Visible light exposure of **M1** crystals, resulting in nearly colorless crystals, gives the dehydrated 15-*trans* structure (2.11 Å resolution). (*d*) Overlay of the two low (cyan), and even lower (green), p*K*_a_ 15-*trans* structures.

We next sought to understand the mechanism of photoswitching in hCRBPII using strategies previously developed for hCRABPII by recapitulation of the photoswitching process in single crystals to structurally monitor the process at atomic resolution. To this end, **M1** was incubated with retinal for 2 h and was subsequently subjected to crystallization in the dark. Retinal-bound **M1** crystals were also obtained in ambient light for comparison. The crystals grown in the dark had a blue tint, while the crystals grown in light were faintly yellow-tinted, consistent with the color of the SB (Supplementary Fig. S5). Exposure of the blue-tinted crystals grown in the dark to visible light resulted in an observable conversion to yellow-tinted crystals (Supplementary Fig. S5). Exposure of either crystal state to UV light resulted in crystals that were dark blue in color. Structural determination of crystals not exposed to UV light all showed a binding pocket similar to that seen in previous retinal-bound structures, however with the *trans*-imine isomer common to Gln4 mutants (Fig. 3 ▸*a*). The *trans*-imine shows the imine N atom surrounded by hydrophobic residues, consistent with its lower p*K*_a_ of 3.3.

On the other hand, crystals irradiated with UV light resulted in retinal-bound structures with a *cis*-iminium isomer, similar to that seen in hCRABPII (Fig. 3 ▸*b*). Also consistent with hCRABPII, the *cis*-iminium makes an NH–π interaction with Trp106, which we surmise is responsible for the higher p*K*_a_ of the *cis*-imine form. Besides the isomerization of the imine, two other structural changes were also observed. A rotation of the Gln38 side chain was seen, resulting in the loss of two ordered water molecules that bridge Gln38 and Gln128 (Fig. 3 ▸ and Supplementary Fig. S1). In addition, an acetate moiety was found to make hydrogen bonds with Glu72, Trp107 and Tyr60. An almost identical bound acetate is seen in many retinal-bound hCRBPII variants, including the parent mutant Q108K:K40L:T51V:Y19W:T53C:R58W:T29L (PDB entry 4efg). Although the rationale behind the movement of Gln38 is unclear, the presence of the bound acetate is correlated with the addition of the extra positive charge in the binding site from the protonation of the imine.

More intriguing was the additional photoconversion to the colorless crystal form on exposure to visible light. We surmise that the loss of color may be due to an even larger acidic shift in the p*K*_a_ of the iminium beyond 3.3. Structure determination of either dark-adapted or visible light-irradiated crystals both showed the *trans*-imine, with every other residue in the binding pocket essentially identical. The only difference was found in the ordered solvent. In the dark-adapted, blue tinted crystals, two water molecules are seen bridging Gln38 and Gln128 (Fig. 3 ▸*b* and Supplementary Fig. S4), a water network recapitulated in many retinal-bound variants (see, for example PDB entries 4ruu or 4ede; Supplementary Fig. S1; Wang *et al.*, 2012 ▸). Nonetheless, in the light-irradiated, almost colorless crystals, both water molecules have disappeared from the electron density (Figs. 3 ▸*c* and 3 ▸*d* and Supplementary Fig. S4). We hypothesized that perhaps this change in solvent may cause an even further acidic shift in the p*K*_a_ of the crystalline form, resulting in the colorless crystals. This would be surprising as these water molecules are not directly interacting with the Schiff base and are in fact more than 7 Å from the N atom (Supplementary Fig. S6). To test this hypothesis, we mutated Gln38 to Leu, resulting in the Q108K:K40L:T51V:Y19W:T53C:R58W:T29L:Q4A:Q38L (**M2**) hCRBPII variant. Upon complexation with retinal, the PSB of this mutant shows a blue shift in its absorption spectrum to 550 nm (Supplementary Fig. S7). Over time, the SB absorption at 360 nm grows, correlated with a loss in PSB absorbance at 550 nm, which we assume is the thermal imine *cis*/*trans* isomerization previously identified. Titration of the resulting low-p*K*_a_ form indicates a p*K*_a_ significantly lower than 3.3 for this variant, although a precise value could not be obtained due to protein instability at extremely low pH. The value of the high-p*K*_a_ form could not be accurately measured due to the combination of the slow binding of the ligand to the protein and the relatively fast conversion of the *cis* to *trans* isomer (Supplementary Fig. S7). The apparent low p*K*_a_ is consistent with the concept that changes in hydration in this region can have a significant effect on the p*K*_a_ of the *trans*-iminium.

As expected, the structure of retinal-bound **M2** shows the *trans*-imine, as predicted for a Gln4 mutant. It also shows that both of the ordered water molecules that were interacting with Gln38 and Gln128 are lost, while all the other residues as well as the conformation of retinal are identical in **M1** and **M2** (Supplementary Fig. S8). Together, these observations are consistent with the idea that solvent *inside* the binding cavity of a protein can have substantial effects on the p*K*_a_ of amino-acid side chains or other bound functional groups, even though they are significantly separated in space. It also suggests that unexpected mechanisms may be at play in the modulation of p*K*_a_ inside protein binding cavities or active sites that involves the rearrangement of the interior solvent in the cavity.

Using a combination of UV measurements and high-resolution crystallographic data, three intermediates are identified in the **M1** photocycle (Fig. 3 ▸). These intermediates all have distinct iminium p*K*_a_ values determined by the protein ‘electrostatic microenvironment’ surrounding the ionizable moiety. These results suggest that a protein environment’s well known ability to have large multi-unit effects on the p*K*_a_ of internal moieties can be dynamic, where relatively modest structural changes can lead to dramatic differences in the p*K*_a_ and therefore any activity that relies on proton transfer. These results highlight the remarkable power and ability of a protein to control its interior electrostatic microenvironment. The functional significance of electrostatics in enzyme activity, originally introduced by Warshal, has been extensively studied by the Boxer laboratory and others (Fried & Boxer, 2017 ▸; Léonard *et al.*, 2021 ▸; Warshel *et al.*, 2006 ▸). Here, we show the dynamic nature of this electrostatic environment, driven by relatively modest structural changes. Together, these insights suggest that protein dynamics may be correlated to changes in interior electrostatics, partly explaining the critical role that motion plays in protein function.

## 4. Conclusions

We describe a new engineered system to mimic a retinal photocycle similar to that seen in rhodopsin proteins. In this system, similar to some rhodopsin family members, photoisomerization results in switching between two distinct p*K*_a_ regimes. Further, high-resolution structural data depict the important role of water molecules on the p*K*_a_ of the retinylidene. This engineered system illuminates novel biophysical properties related to protein–chromophore interactions and can also be used as a new protein target for the design of new photoswitchable fluorescent protein tags.

## Supplementary Material

PDB reference: human retinol-binding protein II, **M2** mutant, 7lhm

PDB reference: **M1** mutant, dark, 7lhn

PDB reference: light-irradiated, 7lho

PDB reference: UV-irradiated, 9pn1

Supplementary Figures. DOI: 10.1107/S2059798326003839/myl5001sup1.pdf

## Figures and Tables

**Table 1:** PCR conditions for amplification of hCRBPII mutants

Reactant	Volume
DNA template	70 ng (*x* µl)
Forward primer	10 pmol (1 µl)
Reverse primer	10 pmol (1 µl)
10 m*M* dNTP	1 µl
10× Pfu reaction buffer	5 µl
PfuTurbo DNA polymerase (2.5 U µl^−1^)	1 µl
Water (PCR grade)	(50 − *x* − 9) µl

**Table 2:** PCR program

Cycles	Temperature (°C)	Time (min:s)
1×	94	3:00
20×	95	0:20
	*T*_m_ − 5	0:55
	72	3:30
1×	72	10:00
1×	4	Hold

**Table 3:** Primers

Q108K	
Forward	5′-CCGCGGCTGGAAG**AAG**TGGATTGAGGGGG-3′
Reverse	5′-CCCCCTCAATCCA**CTT**CTTCCAGCCGCGG-3′
K40L	
Forward	5′-CTCACTCAGACG**CTG**GTTATTGATCAAGATGG-3′
Reverse	5′-CCATCTTGATCAATAAC**CAG**CGTCTGAGTGAG-3′
T51V	
Forward	5′-GGTGATAACTTCAAG**GTA**AAAACCACTAGCAC-3′
Reverse	5′-GTGCTAGTGGTTTT**TAC**CTTGAAGTTATCACC-3′
T53C	
Forward	5′-CAAGACAAAA**TGC**ACTAGCACATTCCG-3′
Reverse	5′-CGGAATGTGCTAGT**GCA**TTTTGTCTTG-3′
R58W	
Forward	5′-CTAGCACATTC**TGG**AACTATGATGTG-3′
Reverse	5′-CACATCATAGTT**CCA**GAATGTGCTAG-3′
Y19W	
Forward	5′-CTTTGAGGGC**TGG**ATGAAGGC-3′
Reverse	5′-GCCTTCAT**CCA**GCCCTCAAAG-3′
T29L	
Forward	5′-GATTTTGCC**CTG**CGCAAGATTGC-3′
Reverse	5′-GCAATCTTGCG**CAG**GGCAAAATC-3′
Q4R	
Forward	5′-GACGAGGGAC**AGG**AATGGAACC-3′
Reverse	5′-GGTTCCATT**CCT**GTCCCTCGTC-3′
Q4A	
Forward	5′-GACGAGGGAC**GCG**AATGGAACC-3′
Reverse	5′-GGTTCCATT**CGC**GTCCCTCGTC-3′
K40L:Q38L	
Forward	5′-CGTCTCACT**CTG**ACG**CTG**GTTATTG-3′
Reverse	5′-CAATAAC**CAG**CGT**CAG**AGTGAGACG-3′

**Table 4:** Anion-exchange FPLC program

Description	Salt content	Volume (ml)	Time (min)
Isocratic flow	0% 1 *M* NaCl	70	17.5
Linear gradient	4% 1 *M* NaCl	10	2.5
Isocratic flow		20	5
Linear gradient	8% 1 *M* NaCl	10	2.5
Isocratic flow		15	3.75
Linear gradient	15% 1 *M* NaCl	15	3.75
Isocratic flow		20	5
Linear gradient	30% 1 *M* NaCl	10	2.5
Isocratic flow		10	2.5
Linear gradient	100% 1 *M* NaCl	10	2.5
Isocratic flow		25	6.25
Linear gradient	0% 1 *M* NaCl	25	6.25
Total		240	60

**Table 5:** Crystallographic data and refinement statistics Values in parentheses are for the outer shell.

	**M1**			**M2**
	Dark, all-*trans*	Light-exposed, 15-*trans*	UV-irradiated, 15-*cis*	15-*trans*
Wavelength (Å)	1.127	1.127	1.127	1.127
Resolution range (Å)	27.66–1.40 (1.45–1.40)	29.48–2.11 (2.185–2.110)	21.28–1.261 (1.306–1.261)	28.61–1.401 (1.451–1.401)
Space group	*P*1	*P*1	*P*1	*P*1
*a*, *b*, *c* (Å)	30.842, 35.918, 64.062	30.755, 35.773, 64.153	30.923, 36.038, 64.026	30.991, 36.091, 64.047
α, β, γ (°)	85.950, 86.434, 65.334	86.180, 86.478, 65.224	86.203, 86.555, 65.059	86.107, 86.561, 65.368
Molecules per asymmetric unit	2	2	2	2
Total reflections	223152	499611	712213	500614
Unique reflections	41917 (4314)	12563 (1254)	58186 (5523)	47394 (4469)
Completeness (%)	85.44 (87.77)	88.28 (88.93)	86.21 (81.77)	95.93 (91.28)
Average *I*/σ(*I*)	14.2	13.0	10.1	12.9
CC_1/2_	0.910	0.926	0.890	0.915
Reflections used in refinement	41915 (4314)	12558 (1245)	58153 (3892)	47382 (4467)
Reflections used for *R*_free_	2059 (206)	1265 (125)	1960 (140)	1957 (194)
*R*_work_ (%)	0.1859 (0.2141)	0.1825 (0.1974)	0.1937 (0.2255)	0.1962 (0.2761)
*R*_free_ (%)	0.2085 (0.2534)	0.2671 (0.2972)	0.2206 (0.2812)	0.2090 (0.2756)
R.m.s.d. from ideal values				
Bond lengths (Å)	0.006	0.009	0.008	0.007
Bond angles (°)	0.96	1.19	1.15	1.01
Average *B* factor (Å^2^)	12.34	21.28	18.52	17.34
No. of water molecules	101	55	232	95
PDB code	7lho	7lhn	9pn1	7lhm
