# Supplementary material for: Photoisomerization detected in a fully wavelength-tunable rhodopsin mimic system
Source: Acta Crystallogr D Struct Biol. 2026 May 27;82(Pt 6):664–71. doi: 10.1107/S2059798326003839 (PMC13224928; doi:10.1107/S2059798326003839)
Supplement: Supplementary file 1 [file d-82-00664-sup1.pdf]

STRUCTURAL
BIOLOGY

Volume 82 (2026)

Supporting information for article:

Photoisomerization detected in a fully wavelength-tunable rhodopsin mimic system

Nona Ehyaei, Courtney Bingham, Katelyn Silva, Zahra Nossoni, Hadi Nayebi Gavgani, Meisam Nosrati, Joelle Eaves, Mustapha Akhdar, Chrysoula Vasileiou, Babak Borhan and James H. Geiger

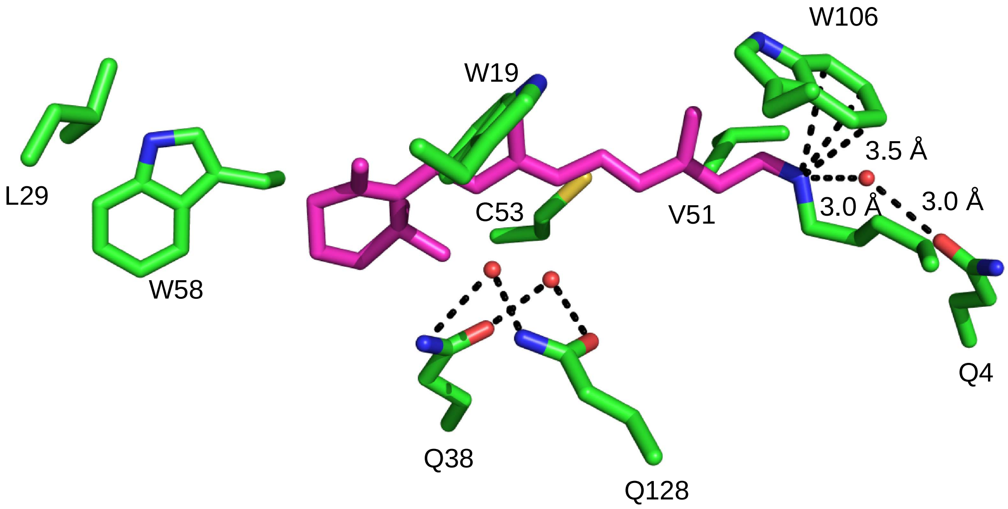

Figure S1 Structure of retinal-bound hCRBPII Q108K:K40L:T51V:T53C:Y19W:R58W:T29L (PDBID 4EDE). Note the PSB is stabilized through a water mediated hydrogen bond involving Gln4 and NH-π interaction with Trp106.

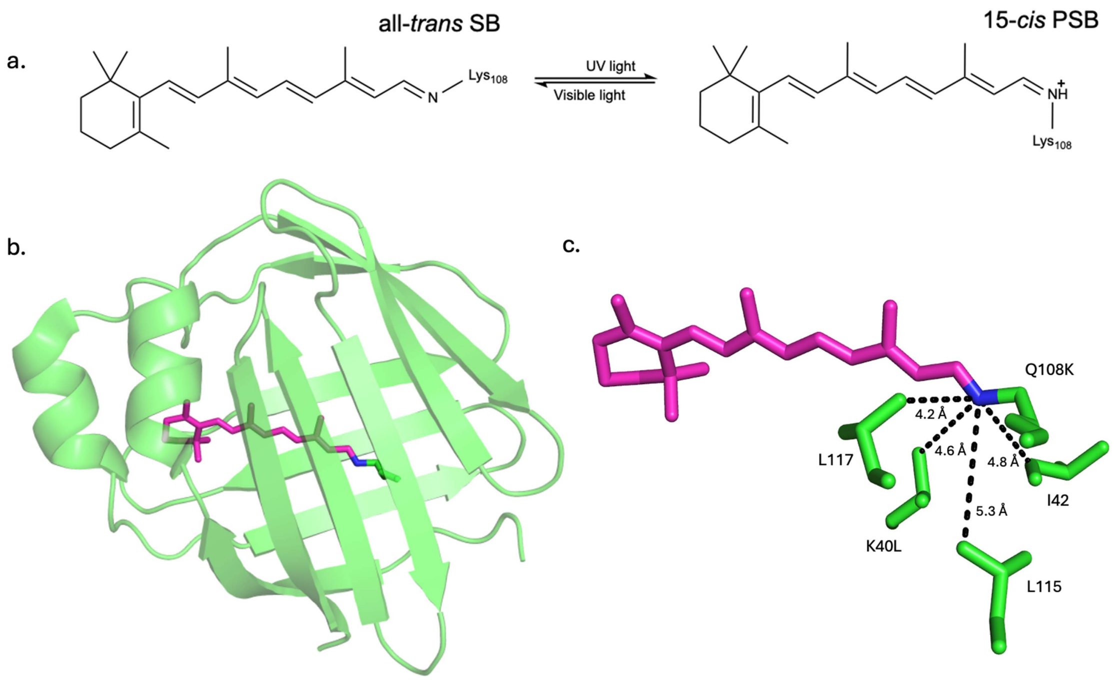

Figure S2 a. Scheme of all-trans to 15-cis imine isomerization. b. Structure of retinal-bound hCRBPII Q108K:K40L:T51V:T53C:Y19W:R58W:T29L:Q4R (PDBID 4EEJ) showing the all-trans retinal covalently bound to Lys 108 in the binding pocket. c. Retinal bound to Lys 108 in the all-trans isomer. K40L, L117, L115, and I42 are each within ~ 5 Å of the imine nitrogen, comprising a hydrophobic binding pocket that depresses the pK_a of the iminium. (PDBID 4EEJ).

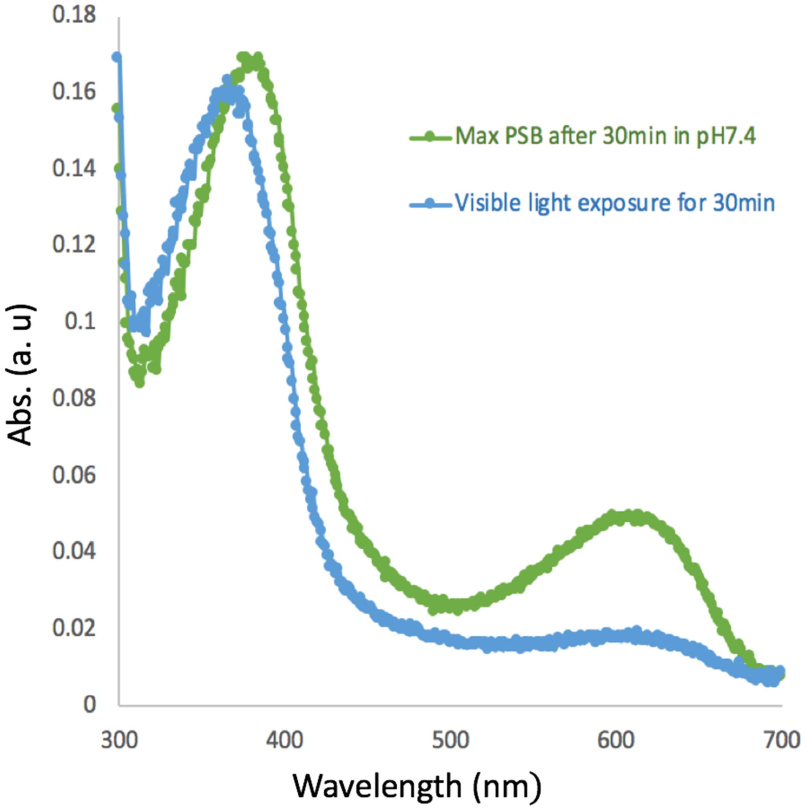

Figure S3 The visible light photo-conversion of the Schiff base from a higher pK_a (PSB) to lower pK_a (SB) at pH = 7.4 in retinal-bound-M1.

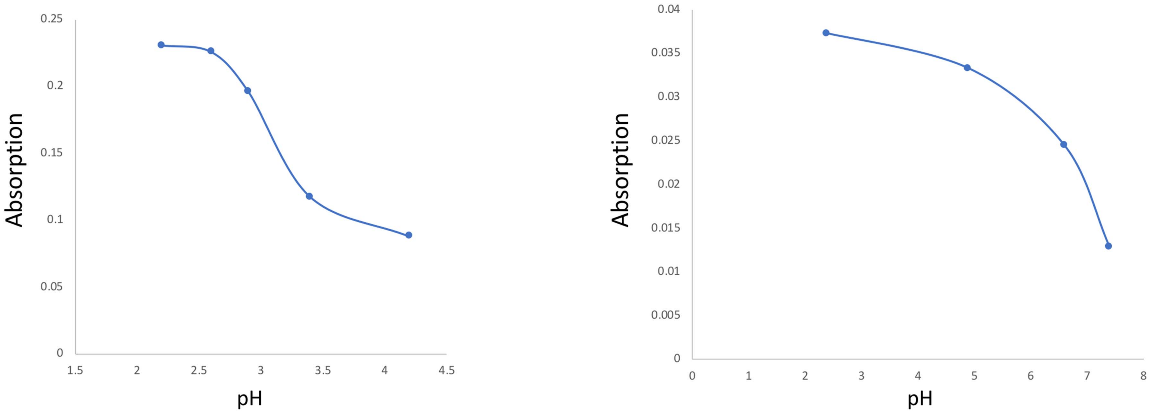

Figure S4 pH titrations for the trans (pK_a 3.3, left) and cis (pK_a 7, right) isomers of **M1**-bound retinal. A complete pH curve was not possible due to protein instability at higher pH.

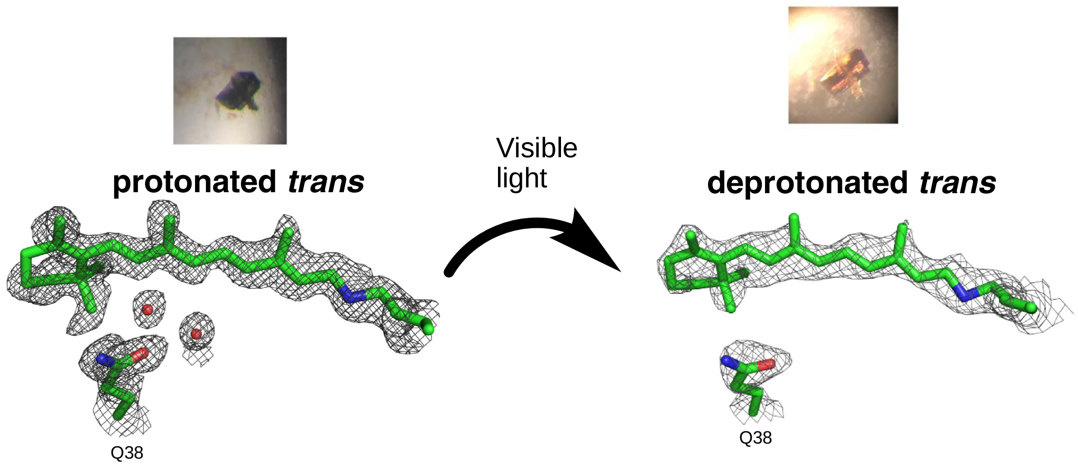

Figure S5 Visible light exposure of the low pK_a *trans* iminium isomer of retinal-bound Q108K:K40L:T51V:T53C:Y19W:R58W:T29L:Q4A (**M1**) hCRBPII causes "photodehydration" of two water molecules, leading to the color change in the crystal from dark blue to tinted yellow due to the shift to an even lower pK_a at pH of 4. Electron density (grey mesh) contoured at 1σ.

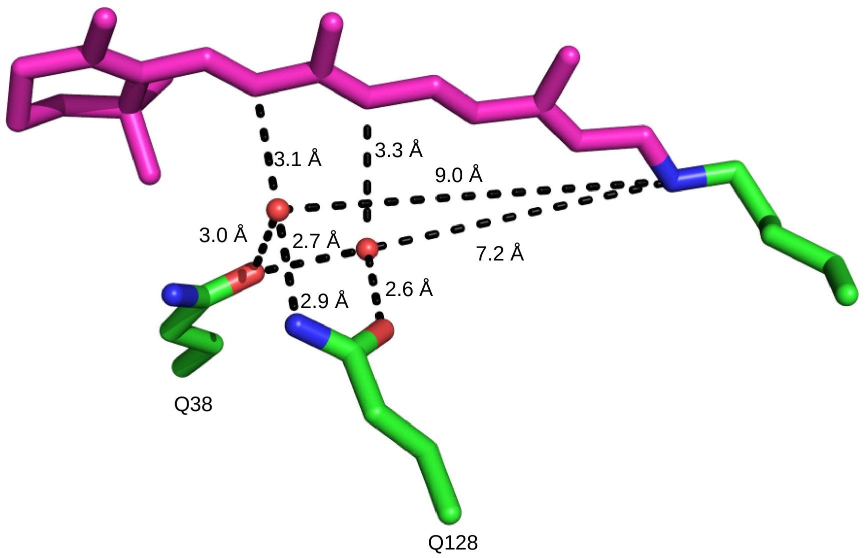

Figure S6 Structure of the retinal-bound Q108K:K40L:T51V:T53C:Y19W:R58W:T29L:Q4A hCRBPII variant (**M1**). The two water molecules bridging Gln 38 and Gln 128 are more than 7 Å from the Schiff base but are within 3.1-3.3 Å of the polyene.

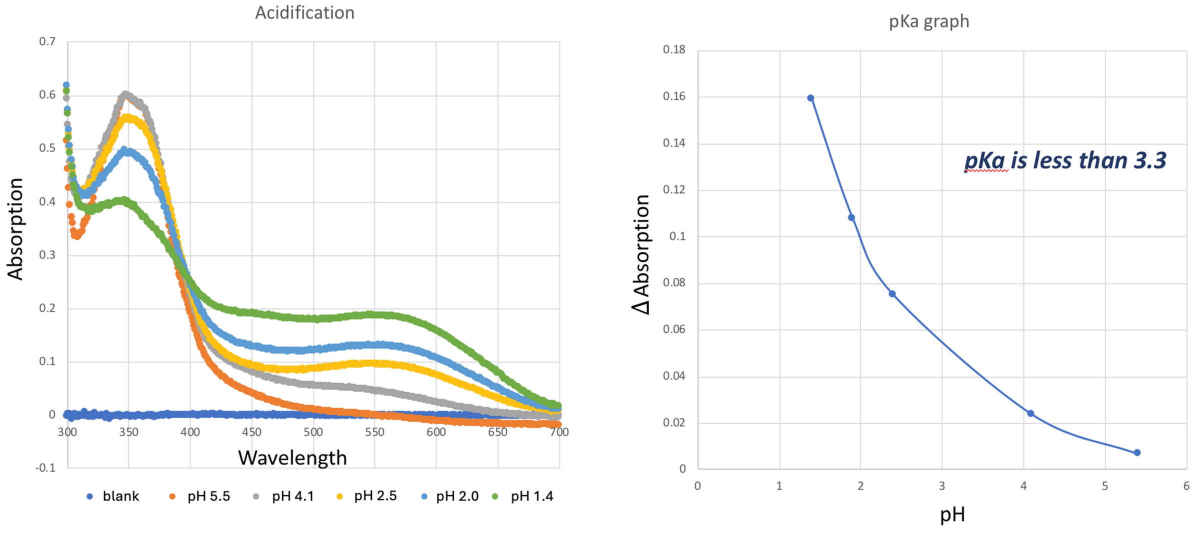

Figure S7 The pK_a measurement for **M2**. The pK_a graph demonstrates that the pK_a for this mutant is lower than 3.3. A complete pK_a curve was not possible due to protein instability at very low pH.

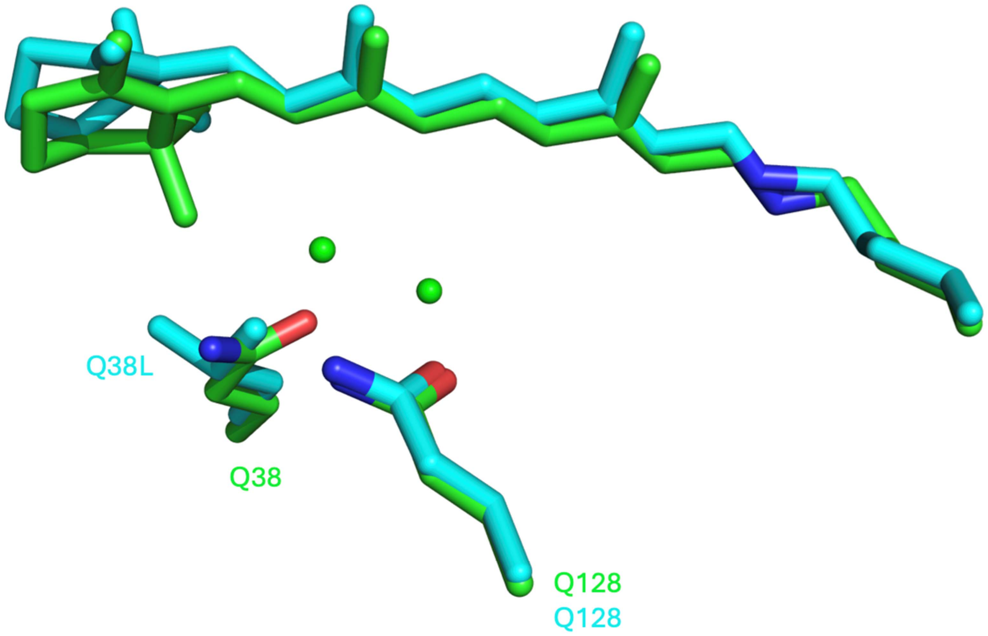

Figure S8 Retinal-bound Q108K:K40L:T51V:T53C:Y19W:R58W:T29L:Q4A **M1** (green) and Q108K:K40L:T51V:T53C:Y19W:R58W:T29L:Q4A:Q38L **M2** (cyan) hCRBPII variant structures overlayed. The only difference other than the Q38L mutation is the loss of the two water molecules, which leads to a much lower pK_a, similar to that seen with visible light-irradiated **M1**.
